# Roles of the DYRK Kinase Pom2 in Cytokinesis, Mitochondrial Morphology, and Sporulation in Fission Yeast

**DOI:** 10.1371/journal.pone.0028000

**Published:** 2011-12-12

**Authors:** Pengcheng Wu, Ran Zhao, Yanfang Ye, Jian-Qiu Wu

**Affiliations:** 1 Department of Molecular Genetics, The Ohio State University, Columbus, Ohio, United States of America; 2 Department of Molecular and Cellular Biochemistry, The Ohio State University, Columbus, Ohio, United States of America; Duke University Medical Center, United States of America

## Abstract

Pom2 is predicted to be a dual-specificity tyrosine-phosphorylation regulated kinase (DYRK) related to Pom1 in *Schizosaccharomyces pombe*. DYRKs share a kinase domain capable of catalyzing autophosphorylation on tyrosine and exogenous phosphorylation on serine/threonine residues. Here we show that Pom2 is functionally different from the well-characterized Pom1, although they share 55% identity in the kinase domain and the Pom2 kinase domain functionally complements that of Pom1. Pom2 localizes to mitochondria throughout the cell cycle and to the contractile ring during late stages of cytokinesis. Overexpression but not deletion of *pom2* results in severe defects in cytokinesis, indicating that Pom2 might share an overlapping function with other proteins in regulating cytokinesis. Gain and loss of function analyses reveal that Pom2 is required for maintaining mitochondrial morphology independently of microtubules. Intriguingly, most meiotic *pom2Δ* cells form aberrant asci with meiotic and/or forespore membrane formation defects. Taken together, Pom2 is a novel DYRK kinase involved in regulating cytokinesis, mitochondrial morphology, meiosis, and sporulation in fission yeast.

## Introduction

Dual-specificity tyrosine-phosphorylation regulated kinases (DYRKs), a family of evolutionarily conserved protein kinases, are known for their capability of autophosphorylating tyrosine residues in their own activation loops and phosphorylating serine/threonine residues on exogenous substrates [1]–[3]. DYRK proteins have functions in diverse biological processes. Based on the homology within the kinase domain, the DYRK family can be grouped into three subfamilies: DYRK kinases, homeodomain-interacting protein kinases, and pre-mRNA processing protein 4 kinases [4].

The functions of several DYRK kinases in yeasts, *Caenorhabditis elegans*, *Drosophila*, and mammals have been unraveled, and accumulating evidence suggests that DYRK kinases function in cell proliferation and cell differentiation [5]. *S. pombe* Pom1 and *S. cerevisiae* Yak1 are the two best characterized DYRK members. Pom1 provides positioning cues for polarized growth and cytokinesis [6]–[9], controls bipolar cell growth [10], and regulates mitotic entry by sensing the cell size via a Pom1 gradient and kinase Cdr2-dependent nodes on the plasma membrane [11]–[15]. Yak1 (the founding member of the DYRK family), on the other hand, is a growth inhibitor by negatively regulating the Ras/cAMP and TOR (target of rapamycin) pathway under nutritional stress [16]–[18]. In *C. elegans*, Mbk1 and Mbk2 are two identified DYRK family members. Mbk1 probably plays a role in neuronal development as overexpression of Mbk1 leads to chemotaxis and olfaction defects [19]. Mbk2 is essential and functions by stimulating degradation of maternal proteins during embryonic development [19], [20]. In *Drosophila* and humans, DYRK kinases have essential functions in postembryonic neurogenesis [21], [22]. In humans, DYRK1A, the best-characterized mammalian DYRK, is implicated in learning defects and is mapped to the “Down syndrome critical region” of chromosome 21 [23], [24] with septin 4 as its specific target [25].

Cytokinesis in fungi, amoebas, and animal cells requires coordination of four key events: cleavage-site selection, assembly of the contractile ring, constriction and disassembly of the contractile ring, and targeted membrane fusion and cell separation [26]–[28]. *S. pombe* has emerged as a very attractive model system for the analysis of cytokinesis [29]–[31]. Anillin Mid1 specifies the division site by a balance of positive and negative signaling cues on the plasma membrane including the inhibitory Pom1 gradient [13], [32]. An actomyosin contractile ring is essential for cytokinesis in *S. pombe* as in animal cells. Mid1 recruits myosin-II, formin, and other proteins to assemble cytokinesis nodes on the equatorial plasma membrane [33]–[35]. The nodes condense into a compact contractile ring by the end of anaphase A through interactions between myosin-II and actin filaments nucleated by formin [36], [37]. The ring matures by recruiting more proteins during anaphase B, which is regulated by the septation initiation network [38]–[40]. After spindle breakdown at the end of anaphase B, ring constriction, targeted membrane fusion, and septum formation partition a mother cell into two mononuclear daughter cells [28].

Mitochondria are dynamic double-membrane organelles with highly plastic morphology and specialized functions. The inner mitochondrial membrane (IMM) and outer mitochondrial membrane (OMM) separates mitochondrial matrix from cytosol, which facilitates the compartmentalization of multiple reactions housed by mitochondria including oxidative phosphorylation and ATP generation, lipid oxidation, calcium homeostasis and apoptosis [41]. Fusion and fission, two antagonizing processes, operate in a coordinated manner to drive the constant morphological changes of mitochondria to form either interconnected tubules or small vesicle units [42]–[44]. Mitochondrial fusion and fission have been investigated extensively in budding yeast. Dynamin-related GTPases Fzo1 and Mgm1 are key proteins for the mitochondria fusion machinery that functions by nucleotide-regulated self-assembly and hydrolysis [41], [44]. Dynamin-related Dnm1 GTPase, integral membrane protein Fis1 as well as its binding partners Mdv1 and Caf4 are proteins important for fission [42]. In addition to remodeling through fusion and fission, mitochondria can travel long distances within cells along the cytoskeleton tracks [44]. The dynamic nature of mitochondrial morphology and distribution fine-tunes mitochondrial functions to respond efficiently to the intracellular demands and outside signals.

Sporulation is a developmental process during nutrient starvation. In fission yeast, the assembly of the double-layered forespore membranes (FSM) coordinates tightly with nuclear divisions during meiosis II for proper segregation and packaging of haploid chromosomes into individual spores [45]–[47]. FSM initiates in the vicinity of the spindle pole bodies (SPBs) at prophase II [48]–[50] to form a cap-like structure surrounding SPBs and then expands by fusing with membrane vesicles derived from endoplasmic reticulum/Golgi as nuclei divide during anaphase II [47], [49], [51]–[54]. During FSM expansion, membrane associated complexes including septins and leading edge proteins are required for shaping the ever-expanding FSM before closure [55], [56]. A set of mutants defective in meiosis or sporulation have been isolated and analyzed in *S. pombe* [57]–[59]. However, only a few genes have been characterized in *S. pombe* related to FSM assembly [45].

Pom2 (SPAC16C9.07/Ppk5/Mug189) is predicted to be a DYRK kinase in *S. pombe* in addition to the well-studied Pom1. However, no biological functions have been reported for Pom2 except a systematic study of fission yeast protein kinases [60]. Here we characterized Pom2's functions during fission yeast life cycles. Our results show that Pom2 localizes to the division site during cytokinesis and mitochondria throughout the cell cycle. Pom2 is required for maintaining the morphology and distribution of mitochondria. Furthermore, we find that Pom2 is essential for both meiosis and FSM assembly in *S. pombe*.

## Results

### The kinase domain of Pom2 functionally complements that of Pom1 in division-site placement

**Figure 1:**
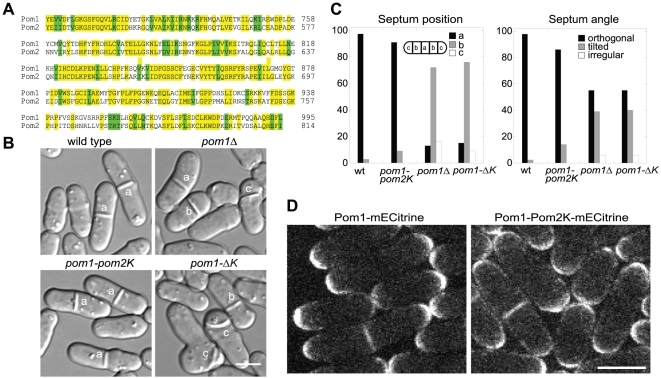
The Pom2 kinase domain complements the Pom1 kinase domain. (A) Sequence alignment of the kinase domains of *S. pombe* DYRK family members Pom1 and Pom2. Protein sequences were aligned using the Vector NTI program. Identical and similar (D/E, I/L/V, K/R, N/Q, S/T) amino acids are shaded in yellow and green, respectively. (B) Differential-interference-contrast (DIC) images and (C) quantification of septum positioning defects in *pom1* mutants. Cells of wild type (strain JW81), *pom1Δ* (JB109), *pom1-pom2K* (JW1802), and *pom1-ΔK* (JW1745) were grown at 25°C. (C) >200 cells for each strain were quantified as described [6]. Septum position was scored by dividing cells into five equal sections (see examples in B). Septum angle was relative to the cell long axis. Septa with angles of 80° to 90° were counted as orthogonal, and septa with smaller angles as tilted. Septa with very aberrant appearance or placed longitudinally were counted as irregular. (D) Localization of Pom1-mECitrine (strain JW1836) and Pom1-pom2K-mECitrine (JW1837). Bars, 5 µm.

Sequence alignment reveals that DYRK kinases Pom1 and Pom2 share 55% identity and 75% similarity in their kinase domains (Figure 1A) but are divergent outside the kinase domain. Since Pom1 relies on its kinase activity for its normal localization to the plasma membrane to perform its biological functions [14], [61], we hypothesized that Pom2 may share partial functions with Pom1. Functional complementation of the full-length proteins was examined first. Overexpression of *pom2* induced by *41nmt1* promoter in EMM5S for 24 h or longer could not rescue the phenotype of *pom1Δ* cells, nor did *pom2Δ* worsen the defects of *pom1Δ* (data not shown). Thus, we concluded that Pom2 and Pom1 have no obvious redundant functions.

Next we focused on the function of the kinase domain. A chimera strain *pom1-pom2K* was created by replacing Pom1 kinase domain with that of Pom2 (see Materials and Methods). As shown in Figure 1B and 1C, *pom1-ΔK* resembled *pom1-Δ1* [6] with asymmetrically placed septa, consistent with a previous report that the kinase-dead *pom1* mutant resembles the *pom1Δ* mutant [61]. However, the *pom1-pom2K* strain resembled wild-type cells in septum position and orientation (Figure 1, B and C), even when cells were grown at 36°C (data not shown). Then we compared the localization of Pom1-Pom2K and Pom1 tagged with mECitrine at the COOH-terminus under the control of the *pom1* promoter. Pom1-Pom2K was detected at both cell ends (asymmetrically in some cells) and the division site, which was similar to the localization of Pom1 (Figure 1D). Taken together, the kinase domain of Pom2 can substitute for that of Pom1 and they share similar biological functions.

### Pom2 localizes to mitochondria throughout the cell cycle and to the division site during cytokinesis

**Figure 2:**
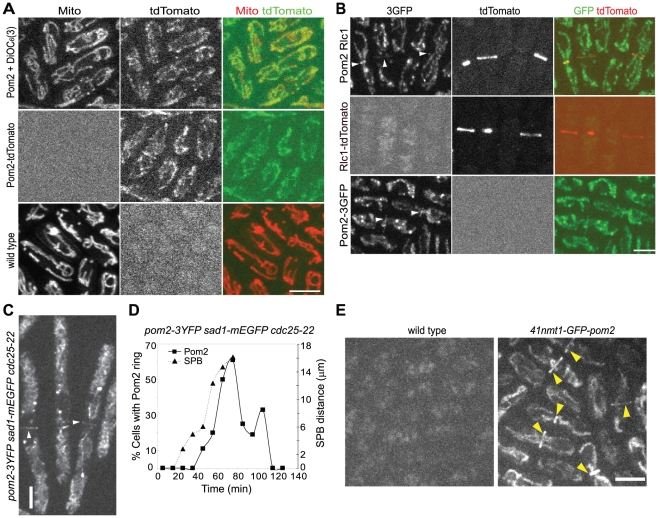
Pom2 localizes to mitochondria throughout the cell cycle and to the division site during cytokinesis. (A) Pom2 localizes to mitochondria. Cells expressing Pom2-tdTomato (strain JW1607) stained with mitochondrial dye DiOC_6_(3) (top) or unstained (middle) and DiOC_6_(3) stained wild-type cells (bottom, strain JW81) were imaged using exactly the same settings. (B) Pom2 co-localizes with Rlc1 in the contractile ring. Cells expressing Pom2-3GFP (bottom, strain JW1605), Rlc1-tdTomato (middle, JW1341), or both (top, JW1714) were imaged using exactly the same settings. Pom2 rings are marked by arrowheads. (C and D) Timing of Pom2 localization to the division site during cytokinesis. Temperature-sensitive *cdc25-22 pom2-3YFP sad1-mEGFP* cells (JW2646) were arrested at late G2 by growing at 36°C for 4 h before release to 25°C at time zero. Samples were taken every ten minutes, imaged with seven Z-sections spaced at 0.8 µm and quantified. (C) Maximum intensity projection of whole cells with Pom2-3YFP ring signals marked by arrowheads. (D) Graph of the time course of the fraction of cells with Pom2-3YFP ring signals (left Y-axis) and the mean spindle pole bodies (SPB) separation (right Y-axis). (E) Mildly overexpressed Pom2 localizes to the contractile ring. Wild type 972 and *41nmt1-GFP-pom2* (JW40) strains were grown in YE5S (repressing condition) at 25°C for 33 h before imaging. Images shown are maximum-intensity projections of 28 Z-sections with 0.2-µm spacing. Arrowheads indicate the contractile rings. Bars, 5 µm.

To gain further insight into Pom2 functions, we monitored the localization of Pom2 throughout the cell cycle using live-cell imaging. Native Pom2 was tagged with 3YFP, 3GFP, or tdTomato under the control of its endogenous promoter. Co-localization analyses with mitochondrion-specific staining dye DiOC_6_(3) indicated that Pom2 located to mitochondria (Figure 2A). The use of wild-type cells stained with DiOC_6_(3) as controls ruled out the possible interferences from bleed-through or autofluorescence (Figure 2A).

A closer examination of the localization pattern identified a small fraction of Pom2 at the division site in cells undergoing cytokinesis (Figure 2B, arrowhead). In cells expressing tdTomato tagged Rlc1 (myosin regulatory light chain) as a contractile-ring marker, Pom2 located to mature and contracting rings (Figure 2B). The ring localization was confirmed in synchronized *cdc25-22 pom2-3YFP* cells after the release from late G2 arrest (Figure 2, C and D). Pom2 began localizing to the contractile ring ∼20 min after SPB separation when the spindle was ∼5 µm. The fraction of cells with a Pom2 ring peaked at ∼60 min after SPB separation when the spindle was 16 µm. To further confirm the ring localization of Pom2, Pom2 was mildly overexpressed using *41nmt1* promoter, which was integrated at the 5′ end of *pom2* together with the *GFP(S65T)* encoding gene. Even under the repressing condition, contractile-ring localization of GFP-Pom2 was clearly observed (Figure 2E). Thus, Pom2 localizes to mitochondria throughout the cell cycle and to the contractile ring during anaphase.

### Involvement of Pom2 in cytokinesis

**Figure 3:**
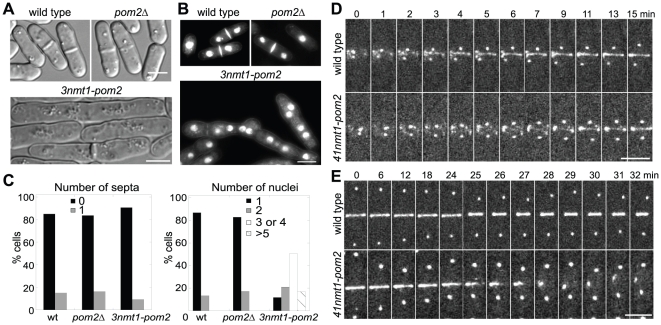
Cytokinesis defects in *pom2* deletion and overexpression cells. (A and B) DIC and fluorescence images of wild type (strain JW81), *pom2Δ* (JW213), and *3nmt1-pom2* (JW216) cells. Cells were grown in EMM5S liquid medium for 30 h at 25°C. (B) Cells were stained with Hoechst to visualize nuclei and Calcofluor to visualize septa. (C) Quantification the septated cells (left) and the numbers of nuclei (right) in strains used in (B) (n>200 for each strain). (D and E) Effects of Pom2 overexpression on contractile-ring formation and constriction. Time course of contractile-ring assembly (D) and constriction (E) during cytokinesis in wild type (JW1568) and *41nmt1-pom2* (JW1626) cells. Cells were induced in EMM5S medium for 24 h. Seven Z-sections spaced at 0.8 µm were taken at each time point. The time point of SPB separation (D) or SPB reaching cell tips (E) is defined as time zero, respectively. Bars, 5 µm.

We hypothesized that Pom2 plays a role in cytokinesis because of its localization to the contractile ring. To test this hypothesis, a *pom2* deletion strain was generated by replacing the entire *pom2* open reading frame with kanMX6. However, the growth rate and morphology of *pom2Δ* were similar to those of wild type in YE5S or EMM5S medium at temperatures from 18 to 36°C (Figure 3, A–C; and data not shown). Even under stress conditions in modified media (EMM5S plus 1M KCl at 18°C; YE5S or EMM5S with 2–10% ethanol, or 1M sorbitol; or non-fermentable carbon sources 3% glycerol, or 2% glycerol plus 3% ethanol), the morphology of *pom2Δ* cells was similar to that of wild type. In addition, no obvious synthetic interactions were detected between *pom2Δ* and mutations in genes involved in various steps of cytokinesis including *mid1*, *plo1*, *cdc4*, *clp1*, *imp2*, *spg1*, *cdc11*, *cdc14*, *cdc16*, *cdc7*, *sid2*, *mid2*, or *spn4*.

However, a role of Pom2 in cytokinesis was discovered in overexpression studies by replacing the *pom2* promoter with *nmt1* promoters at the *pom2* chromosomal locus (Figure 3). Under repressing conditions, most *3nmt1-pom2* cells appeared normal. After shifting to inducing EMM5S medium, cells became highly elongated with no more than one septum. Only ∼9% of *3nmt1-pom2* cells had a septum compared to ∼15% in wild-type and *pom2Δ* cells (Figure 3, A–C). Moreover, multiple nuclei accumulated in *3nmt1-pom2* cells (50% cells with 3 or 4 nuclei and 17% cells with >5 nuclei 30 h after inducing), while only 1 or 2 nuclei were found in wild-type and *pom2Δ* cells (Figure 3, B and C). These results suggest that overexpression of Pom2 may affect: 1) contractile-ring assembly, or 2) contractile-ring constriction and septum formation during cytokinesis. To distinguish these possibilities, we investigated contractile-ring kinetics using Rlc1-3GFP as a ring marker and SPB protein Sad1-mEGFP as an internal cell-cycle clock [38] in cells with mildly over-expressed Pom2 under the inducible *41nmt1* promoter. Observed at 24 h after inducing Pom2, Rlc1 nodes started to appear at the division site ∼10 min before SPB separation, and then the nodes condensed into a compact ring ∼10 min after SPB separation, which was similar to wild type and *pom2Δ* (Figure 3D and data not shown). However, 18 out of 20 cells with the compact rings could not constrict normally and no septum was formed. The ring disassembled immediately upon initiation of ring constriction or collapsed into clumps (Figure 3E). As the kinase domain of Pom2 functionally complements that of Pom1 in division-site placement (Figure 1), we tested whether *pom1* mutants show similar defects in cytokinesis when overexpressed. Consistent with previous studies [11], [12], [61], 24 h after grown in inducing condition (EMM5S), *41nmt1-pom1* expressing cells became longer in cell size at cell division but showed no obvious defect in cytokinesis, while *3nmt1-pom1* expressing cells showed severe defects in cell polarity and formed T-shaped cells (data not shown). Thus, Pom1 and Pom2 do not have overlapping functions in cytokinesis and the cytokinesis defects in Pom2 overexpressing cells are not from the interference with Pom1 function.

Thus, the results of overexpression and loss of function analyses suggest *pom2* may involve in contractile-ring constriction and septum formation during late stages of cytokinesis, but this role is overlapping or redundant with those of other unknown protein(s).

### Normal morphology and distribution of mitochondria depend on Pom2

**Figure 4:**
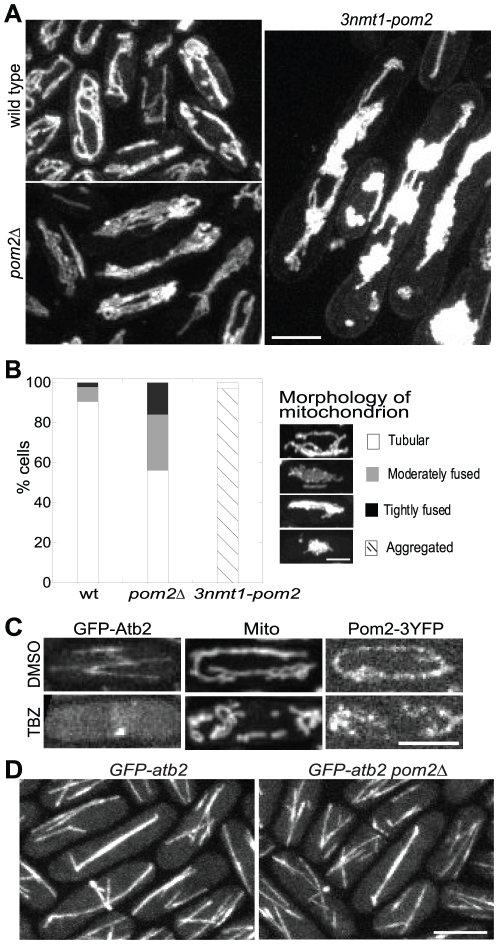
Abnormal mitochondrial morphology in *pom2* mutants and microtubule independence of Pom2 localization to mitochondria. Cells of *3nmt1-pom2* (JW216) were grown in repressing condition and then shifted to inducing condition of EMM5S for 24 h at 25°C. (A) Morphology of mitochondria in wild-type (JW81), *pom2Δ* (JW213), and *3nmt1-pom2* (JW216) cells stained with DiOC_6_(3). (B) Quantification of mitochondrial morphology shown in (A) (n>300 cells for each strain). The morphologies were categorized into four classes: tubular (one to four branched tubules extended from one end of the cell to the other), moderately fused (net-like mitochondria), tightly fused (mitochondria accumulated at one side of the cell along the long axis), and aggregated (mitochondrion formed one or several big aggregates at or close to the cell middle). (C) Microtubule independence of Pom2 localization to mitochondria. DiOC_6_(3) stained wild-type cells (middle, strain JW81), cells expressing tubulin GFP-Atb2 (left, JW1804), or Pom2-3YFP (right, JW1606) were treated with equal amount of microtubule depolymerizing drug TBZ (lower panel) or DMSO (upper panel). (D) Pom2 does not obviously affect microtubule cytoskeleton. Cells expressing GFP-Atb2 in wild type (left, strain JW1804) and in *pom2Δ* (right, strain JW1955) were imaged using the same settings. Bars, 5 µm.

Localization of Pom2 to mitochondria suggests it might regulate mitochondrial morphology and distribution. To elucidate this role, we used mitochondrion-specific stain DiOC_6_(3) to detect mitochondria (Figure 4, A and B). In wild-type cells, tubular-shaped branched mitochondria were distributed throughout the cell. However, the mitochondria in *pom2Δ* mutant were less branched and some of them appeared to be fused together. For quantification purposes, cells were grouped into four classes based on their mitochondrial morphology: tubular, moderately fused, tightly fused, and aggregated as indicated (Figure 4, A and B). In wild type, 90% cells have tubular mitochondria, 8% moderately fused, and 2% tightly fused (n = 450 cells). By contrast, the percentage in *pom2Δ* cells is 55%, 28% and 17% (n = 500), respectively. Overexpression of Pom2 resulted in even more severe defects in mitochondrial morphology and distribution as 97% of *3nmt1-Pom2* cells induced for 24 h at 25°C manifested a mitochondrial aggregate phenotype (Figure 4, A and B).

We next explored the relationship between Pom2 and microtubules, which are crucial for the movement, location, and morphology of mitochondria in fission yeast [62]–[64]. In the presence of 50 µg/ml TBZ or 25 µg/ml MBC, two microtubule depolymerizing drugs, microtubules in the wild-type cells almost disappeared, except for short microtubule stubs near SPBs, and mitochondria appeared fragmented (Figure 4C). Interestingly, Pom2 still localized to mitochondria under the same treatment, although the distribution pattern was not as continuous as the control (Figure 4C), implying that mitochondrial location of Pom2 was microtubule independent. The normal morphology of *pom2Δ* cells suggested the overall microtubule cytoskeleton was normal. Indeed, interphase microtubules, spindle microtubules, and the post-anaphase array appeared normal in *pom2Δ* cells expressing GFP-*atb2* under the *atb2* promoter (Figure 4D). Thus, Pom2 is important for normal morphology and distribution of mitochondria and these roles is microtubule independent.

### Pom2 is critical during the sexual cycle

**Figure 5:**
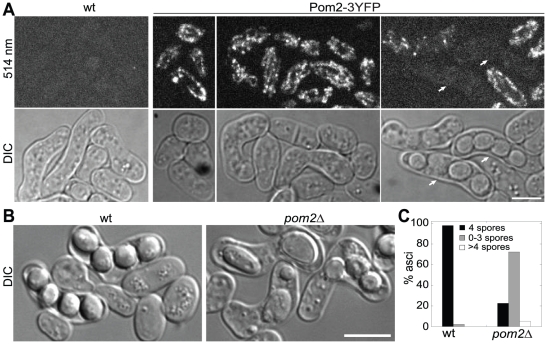
Roles of Pom2 in sporulation. (A) Localization of Pom2-3YFP during mating (the second column), cell fusion (third), and sporulation (fourth). The first column is wild-type control. Pom2-3YFP (JW1606×JW2166) and wild-type strains (JW81×JW729) with opposite mating types were grown in YE5S liquid medium for 12 h and then crossed on SPA5S plate at 25°C. Pairs of fluorescence and DIC images were taken 24 h after the crosses. (B and C) Defective sporulation in *pom2Δ* cells. Wild-type strains JW81 and JW729 (left) or *pom2Δ* strains JW210 and JW213 (right) were crossed on an SPA5S plate at 25°C and examined after 48 h. (B) Asci imaged using DIC. (C) Quantification of spore numbers in each ascus (n>500 asci for each cross). Bars, 5 µm.

Pom2 expression is up-regulated during meiosis [65]. To investigate the possible involvement of Pom2 during mating, meiosis, sporulation, and spore germination, we first investigated the localization of Pom2 during these processes. When *pom2-3YFP* strains of opposite mating types were crossed, Pom2 signal was detected in mitochondria during mating and zygotic stages, resembling the vegetative cells (Figure 5A). However, no Pom2 signal was detected in mature asci with four well formed spores (marked with arrows). Then we crossed *pom2Δ* strains of different mating types to quantify the efficiency of spore formation. 48 h after crossing, only ∼20% of the asci (n = 876) contained four normal-looking spores while the remaining asci contained 0 to 3 or >4 spores, among them 1 or 2 big spores were frequently observed (Figure 5, B and C). In contrast, 98% of the wild-type asci (n = 557) contained four spores (Figure 5, B and C). Even when spores were formed, the viability of *pom2Δ* spores (n = 156) was reduced by half compared to wild type spores by tetrad analysis. Among the spores that did not form colonies, 90% of them were unable to germinate. Together, these data suggest that Pom2 is crucial during the sexual cycle.

**Figure 6:**
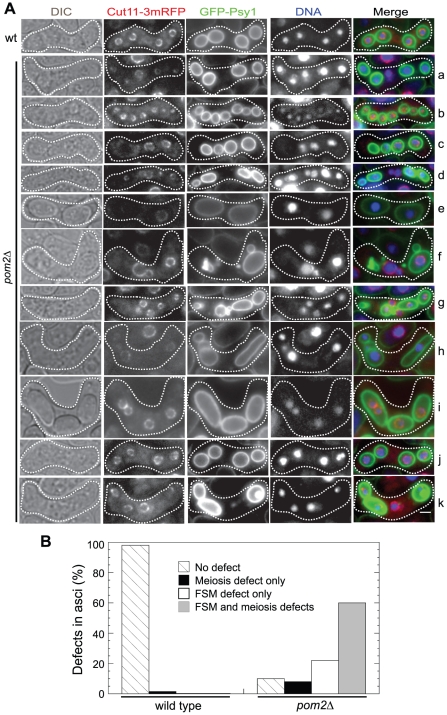
Meiosis and FSM formation defects in *pom2Δ* cells. (A) Representative DIC and fluorescence micrographs showing defects in meiosis and FSM formation in *pom2Δ* cells. *pom2^+^* (JW2619 and JW2620) or *pom2Δ* strains (JW2617 and JW2618) were crossed on SPA5S plates to induce meiosis and sporulation for 11 h before imaging. In merged images, nuclear membrane (Cut11-mRFP), FSM (GFP-Psy1), and nuclear DNA are in red, green, and blue, respectively. Representative images are shown. Images a-g are examples of asci with other than 4 nuclei, h-k are asci with 4 nuclei. (B) Quantification of meiosis and FSM formation defects in *pom2^+^* or *pom2Δ* cells as shown in (A). Asci with 4 well-separated DNA masses and a normal sphere-shaped FSM enclosing each DNA mass were classified as no defect; asci with other than 4 DNA masses but normal FSM morphology with each FSM enclosing one DNA mass, or 4 DNA masses with abnormal nuclei segregation pattern but normal FSM morphology were classified as meiosis defect only; spore membrane formation defect only includes asci with 4 DNA masses but FSM defects: either one or more DNA masses were not enclosed by FSM or two DNA masses were enclosed within one FSM; other asci were classified as defects in both meiosis and FSM formation. Bar, 3 µm.

We attempted to address how Pom2 deletion led to such severe defects in sporulation. As sporulation is a complicated multi-step process in which meiotic nuclear divisions are highly coordinated with the development of FSM, we hypothesized that Pom2 might directly or indirectly participate in FSM formation or meiosis. We used Hoechst to stain DNA together with Cut11-3mRFP [66] and GFP-Psy1 [49] to label nuclear envelope and FSM, respectively (Figure 6, A and B). From the crossing between two *pom2^+^* cells, 98% asci (n = 86 asci) contained 4 well formed spores, and Psy1-labeled FSM enclosed one nucleus in each spore. Of 106 asci from crossing two *pom2Δ* haploid stains of opposite mating types, 61% contained other than 4 DNA masses/nuclei, indicating defects in meiosis (Figure 6A, a–g). In some of these asci the resultant FSMs were misshapen and disoriented or formed spore-like bodies without enclosing nuclei. We reasoned that the defects in FSMs might result from meiotic failure. However, in the 39% asci (n = 106) that contained 4 DNA masses/nuclei, only ∼30% spores had no apparent defects in FSM morphology, the rest displayed defects in failure to encapsulate DNA, contained two nuclei in one spore-like body, or formed misshaped spores (Figure 6A, h–k). Collectively, we find that Pom2 plays important roles in both meiosis and FSM formation.

## Discussion

Although two DYRK kinases Pom1 and Pom2 are 55% identical in their kinase domains and the Pom2 kinase domain functionally complements that of Pom1 in division-site placement, the full-length proteins cannot substitute for each other. Instead, Pom1 and Pom2 play largely non-overlapping roles in fission yeast. The functional specificity might be dictated by their divergent regions outside the kinase domain.

### Roles of Pom2 in cytokinesis

Unlike Pom1 [6], it seems that Pom2 does not have an obvious role in the positioning of the division site. However, several lines of evidence suggest that Pom2 functions in contractile-ring stability/constriction and/or septum formation. First, a small fraction of Pom2 localizes to the contractile ring during anaphase and ring constriction (Figure 2). Second, moderate overexpression of Pom2 causes ring collapse during ring constriction (Figure 3). Third, a high level of Pom2 overexpression leads to multinucleated cells with severe defects in septa formation (Figure 3). However, the role of Pom2 in cytokinesis must be overlapping with those of other proteins because *pom2* deletion cells have no obvious cytokinesis defects under normal or stress conditions. We tried to look for the overlapping genes by genetic interactions of *pom2Δ* with more than a dozen cytokinesis mutants. So far, no synthetic genetic interactions have been found. In the future, synthetic genetic array analysis can be used to uncover other genes playing overlapping functions with Pom2 [67]. In addition, the identification of Pom2 substrates and/or partners in the contractile ring will also help elucidate its roles in contractile-ring stability and constriction.

### Roles of Pom2 in maintaining the morphology of mitochondria

The localization of Pom2 to mitochondria and the mitochondrial defects in *pom2* deletion and overexpression cells indicate that Pom2 is important for mitochondrial morphology. To our knowledge, this is the first time that a DYRK kinase has been observed to be localized to mitochondria and implicated in regulating their morphology. Genes affecting mitochondrial morphology have been reported to play roles in mitochondrial fusion and fission [41], [42], [44], [68]–[71]. The fused and aggregated mitochondria in *pom2* mutants (Figure 4) suggest that Pom2 might be involved in mitochondrial fission instead of fusion. Indeed, in the mating assay [72] of two haploid strains expressing mitochondrial outer membrane protein marker Tom70 (tagged with either cerulean or mECitrine at the COOH-terminus under the native promoter), the fusion of mitochondria after mating occurs as efficiently in *pom2Δ* cells as in wild type (data not shown). However, it seems that COOH-terminally tagged Tom70 is not fully functional. Thus, further experiments are needed to confirm that Pom2 is involved in mitochondrial fission. In addition, more experiments are required to reveal whether Pom2 maintains the morphology of mitochondria by phosphorylating other mitochondrial proteins.

### Roles of Pom2 in meiosis and sporulation

Compared to the mild phenotypes of *pom2* deletion cells during vegetative growth, Pom2 is much more important during meiosis and sporulation (Figures 5 and 6). The observation of abnormal meiosis patterns in *pom2Δ* cells is surprising. Many proteins have been identified to be involved in sporulation. Spo2, Spo13, and Spo15 are important in SPB modification during the early step of sporulation [45], [50], [73]. Spo3, Spo9, Spo14, Spo20, septins [49], [50], [52], [56], [74], and components of the SIN pathway [51], [75]–[79] are crucial for FSM expansion to encapsulate the nuclei after proper SPB modification. None of them has shown obvious meiosis defects. However, Pom2's roles in both meiosis and sporulation are consistent with the upregulation of Pom2 expression during meiosis that peaked at meiosis I [65]. Our data show that Pom2 plays critical roles in both meiosis and FSM formation. It might function in regulating chromosome replication and segregation. Further efforts are needed to decipher the functions at the molecular levels, which will certainly add to our understanding of this unique DYRK kinase in *S. pombe*.

Defects of *pom2* deletion cells in meiosis and sporulation might result from its function in regulating mitochondrial morphology since Pom2 is also enriched in mitochondria during these processes. It has been shown that normal mitochondrial dynamics and functions are important for meiosis, sporulation, and spore viability in both budding and fission yeast [80], [81]. In addition, defects in mitochondrial distribution and functions are linked to human infertility [82]–[85]. Thus, roles of mitochondria in spore and gamete genesis might be conserved during evolution. In *Dictyostelium*, the DYRK kinase YakA is required for the transition between the vegetative growing phase and the developmental phase (including spore formation) under nutrient starvation [86]–[89]. Collectively, it seems that DYRK kinases play crucial roles in both cell proliferation and cell differentiation.

## Materials and Methods

### Yeast strains, growth conditions, genetic and molecular methods

*S. pombe* strains used in this study are listed in Table 1. PCR-based gene targeting was performed as described [90]. All tagged genes are under the control of endogenous promoters and integrated at their native chromosomal loci except those under the control of *3nmt1* and *41nmt1* promoters.

**Table 1:** List of *S. pombe* strains used in this study.

Strain	Genotype	Source/reference
JW40	*h^−^ kanMX6-41nmt1-GFP-pom2*	This study
JW81	*h^−^ ade6-210 ura4-D18 leu1-32*	[38]
JW210	*h^+^ pom2-Δ1::kanMX6 ade6-M210 ura4-D18*	This study
JW213	*h^−^ pom2-Δ1::kanMX6 ade6-M210 leu1-32 ura4-D18*	This study
JW216	*h^−^ kanMX6-3nmt1-pom2 ade6 leu1-32 ura4-D18*	This study
JW237	*h^+^ kanMX6-41nmt1-pom2 leu1-32 ura4-D18*	This study
JW729	*h^+^ ade6-M210 leu1-32 ura4-D18*	[38]
JW1341	*h^−^ rlc1-tdTomato-natMX6 ade6-M210 leu1-32 ura4-D18*	This study
JW1568	*h^−^ rlc1-3GFP-kanMX6 sad1-mEGFP-kanMX6 ade6-M210 leu1-32 ura4-D18*	This study
JW1605	*h^+^ pom2-3GFP-kanMX6 ade6-M210 leu1-32 ura4-D18*	This study
JW1606	*h^+^ pom2-3YFP-kanMX6 ade6-M210 leu1-32 ura4-D18*	This study
JW1607	*h^+^ pom2-tdTomato-kanMX6 ade6-M210 leu1-32 ura4-D18*	This study
JW1626	*kanMX6-41nmt1-pom2 rlc1-3GFP-kanMX6 sad1-mEGFP-kanMX6 leu1-32 ura4-D18*	This study
JW1714	*pom2-3GFP-kanMX6 rlc1-tdTomato-natMX6 ade6-M210 leu1-32 ura4-D18*	This study
JW1745	*h^−^ pom1-ΔK::ura4^+^ ade6-M210 leu1-32 ura4-D18*	This study
JW1802	*h^−^ pom1-pom2K ade6-M210 leu1-32 ura4-D18*	This study
JW1804	*h^−^ Patb2-GFP-atb2-(0.5 kb gap)-kanMX leu1-32 ura4-D18*	[66]
JW1836	*h^+^ pom1-mECitrine-kanMX6 ade6-M210 leu1-32 ura4-D18*	This study
JW1837	*h^−^ pom1-pom2K-mECitrine-kanMX6 ade6-M210 leu1-32 ura4-D18*	This study
JW1955	*Patb2-GFP-atb2-(0.5 kb gap)-kanMX pom2-Δ1::kanMX6 leu1-32*	This study
JW2166	*h^−^ pom2-3YFP-kanMX6 ade6-M210 leu1-32 ura4-D18*	This study
JW2617	*h^−^ cut11-3mRFP-hphMX6 pom2-Δ1::kanMX6 leu1::GFP-psy1 ade6-M210 leu1 ura4?*	This study
JW2618	*h^+^ cut11-3mRFP-hphMX6 pom2-Δ1::kanMX6 leu1::GFP-psy1 ade6-M210 leu1 ura4?*	This study
JW2619	*h^−^ cut11-3mRFP-hphMX6 leu1::GFP-psy1 ade6-M210 leu1 ura4?*	This study
JW2620	*h^+^ cut11-3mRFP-hphMX6 leu1::GFP-psy1 ade6-M210 leu1 ura4?*	This study
JW2646	*pom2-3YFP-kanMX6 sad1-mEGFP-kanMX6 cdc25-22 ade6-M210 leu1-32 ura4-D18*	This study
FY12587	*h^−^ ade6-M210 ura4 leu1::GFP-psy1*	Yeast Genetic Resource Center Japan
JB109	*h^+^ pom1-Δ1::ura4^+^ ade6-M216 ura4-D18*	[6]
JB150	*h^−^ kanMX6-41nmt1-pom1*	Jürg Bähler
JB151	*h^−^ kanMX6-3nmt1-pom1*	[61]
MS1381	*h^−^ cut11-3mRFP-hphMX6 leu1-32 ura4-D18*	[66]
972	*h^−^* wild type	[93]

To construct the *pom1-pom2K* strain for the domain complementation experiments, the Pom1 kinase domain (amino acids 699–995) was replaced by KS-ura4 using PCR-based gene targeting to obtain strain *pom1-ΔK::ura4^+^* [90]. DNA (*pom2K*) encoding the Pom2 kinase domain (amino acids 518–814) was amplified and cloned into the TOPO vector. PCR fidelity was confirmed by sequencing. Then the *ura4^+^* in *pom1-ΔK::ura4^+^* was replaced by the *pom2K* fragment with 66 bp-long homologous sequences to the flanking regions of *pom1* kinase domain. Positive clones were selected on 5-FOA plates and confirmed by PCR. The fusion gene was further confirmed by sequencing.

Cells were re-streaked from −80°C stock, grown 2–3 days on plates, and then inoculated into 5–15 ml YE5S liquid culture as described [91]. Cultures were kept in exponential phase for 36–48 h before microscopy except where specified. Strains with *3nmt1* or *41nmt1* promoter were grown in YE5S medium for at least 24 h, washed 3× with EMM5S, and then induced in EMM5S medium for 12–48 h before microscopy except where noted. Stock solutions of thiabendazole (TBZ; Sigma-Aldrich) and methyl benzimidazole-2-yl carbamate (MBC; Sigma-Aldrich) were made in DMSO at concentrations of 50 mg/ml and 5 mg/ml, respectively. The final working concentrations were 50 µg/ml for TBZ and 25 µg/ml for MBC. Cells were stained with Calcofluor and Hoechst 33342 (bisBenzimide) as described [92]. Stock solution of Hoechst 33342 (Sigma-Aldrich) was made in ddH_2_O at 1 mg/ml and kept in the dark at 4°C.

### Microscopy and data analysis

Cells for microscopy were collected from liquid cultures or plates (for mating and sporulation assays, see below), washed with EMM5S (for mitotic cells) or SPA5S (for sporulating cells), and resuspended in EMM5S or SPA5S for imaging. Live cell microscopy was performed using a thin layer of 100 µl of EMM5S or SPA5S liquid medium containing 20% gelatin (G-2500, Sigma-Aldrich), sealed under a coverslip with Valap, and observed at 23–25°C as described [36], [91]. We used 100×/1.4 NA objective lens (Nikon, Melville, NY) on a spinning disk confocal microscope (Ultra View ERS with CSU22 confocal head, PerkinElmer Life And Analytical Sciences, Inc.) with 440, 488, 514, and 568 nm lasers. Images were acquired with a cooled charge-coupled device camera (ORCA-AG, Hamamatsu). For Figures 1B, 3A, 3B, and 6A, cells or asci were observed using 100×/1.4 NA objective lens on a Nikon Eclipse Ti inverted microscope equipped with a Nikon cooled digital camera DS-QI1 and appropriate filter sets (DIC, DAPI, GFP, Texas Red).

Maximum intensity projections of color images were created using UltraView ERS software. All other image analyses were performed using ImageJ software. Images in figures are maximum-intensity projections of Z-sections spaced at 0.4–0.8 µm unless otherwise noted.

### Staining of mitochondrion

1 ml of exponential phase cells (OD_595_ = 0.3∼0.5) were collected by centrifugation at 5000 rpm for 30 s, and then resuspended in 1 ml EMM5S liquid medium. A final concentration of 0.18 mM DiOC_6_(3) (3,3′-dihexyloxacarbocyanine iodide; Molecular Probes) prepared in ethanol was added to the culture from a 100× stock solution. After 5-min incubation in the dark, cells were collected by centrifugation, washed with 1 ml EMM5S containing 0.1 mM antioxidant n-propyl-gallate once, and then imaged.

### Mating and sporulation assays

1 ml of each haploid strain at exponential growth phase (OD_595_ = 0.3–0.5) was washed in SPA5S liquid medium three times and harvested by centrifugation. The cell pellets were resuspended in 10 µl SPA5S medium and mixed together before plating onto an SPA5S plate at 25°C. Cells were visualized at different time points after the cross. To stain DNA during spore formation, cells scraped from the SPA5S plate were suspended in 1 µg/ml Hoechst 33342 solution and incubated at 25°C for 1 h in the dark before observation.
